# Supplementary material for: Development of a versatile LCM-Seq method for spatial transcriptomics of fluorescently tagged cholinergic neuron populations
Source: J Biol Chem. 2023 Aug 1;299(9):105121. doi: 10.1016/j.jbc.2023.105121 (PMC10477691; doi:10.1016/j.jbc.2023.105121)
Supplement: Supporting Figures S1–S5 [file mmc1.pdf]

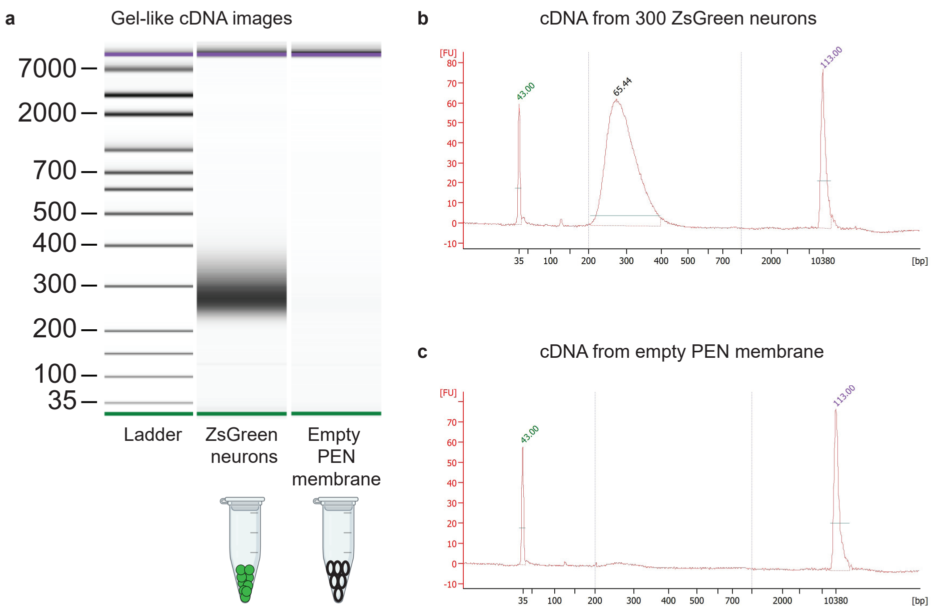

Figure S1: Electropherogram of cDNA samples prepared from 300 microdissected neurons and from tissue-free PEN membrane. a: Gel-like images of cDNA samples generated with the Bioanalyzer High Sensitivity DNA Kit from 300 microdissected ZsGreen neurons and from empty PEN membrane (used as negative control). **b:** Electropherogram of the neuronal cDNA exhibits maximum fluorescent intensity at ~ 280 bp. **c:** This band is absent and the electropherogram contains the size markers only using the tissue-free PEN membrane for RNA isolation and cDNA library preparation.

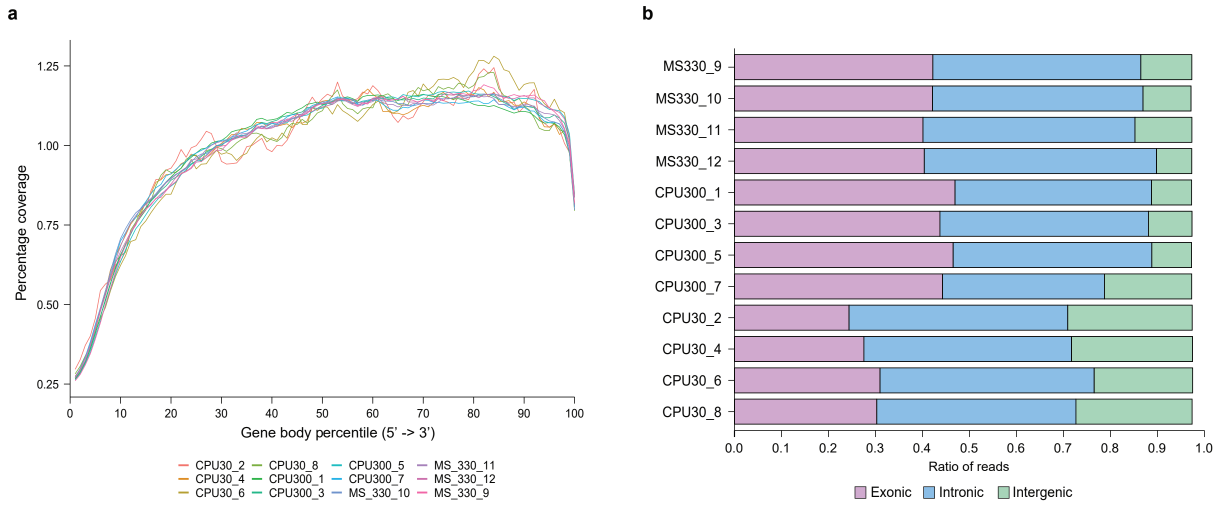

Figure S2: RSeQC analysis of reads. a: Read distribution over gene body plot. **b:** Distribution of transcript-associated reads that map within exons, introns and the genomic space between annotated genes. Note the reduced mapping of reads to exonic sequences and the increase in intergenic reads in the CPU30 samples.

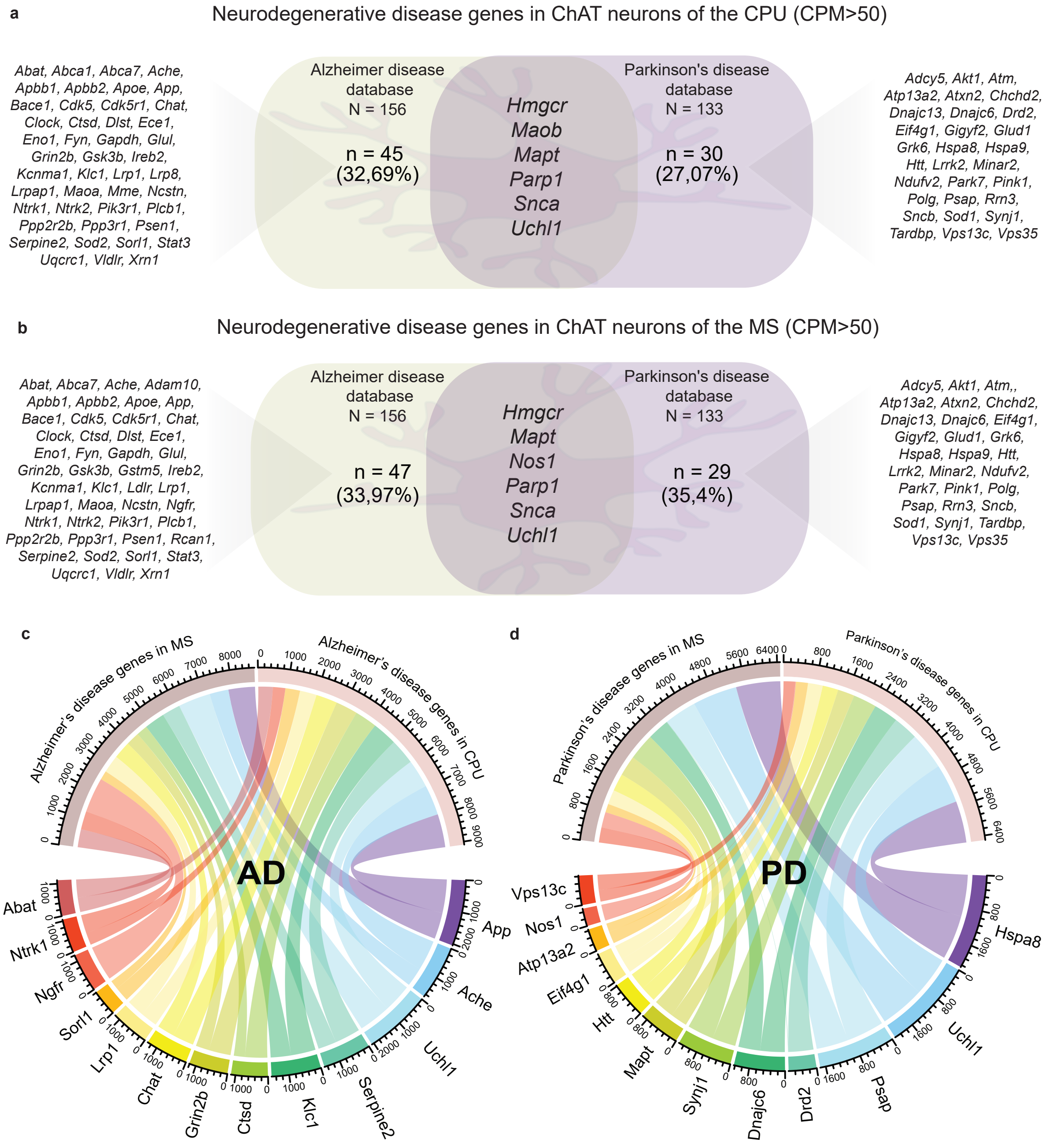

Figure S3: Enrichment of neurodegenerative disease genes in the two spatially defined cholinergic neuron populations. Cholinergic neurons both in the MS and the dorsal CPU highly express genes implicated in Alzheimer's disease (AD) and Parkinson's disease (PD). **a, b:** Venn diagrams reveal that ~26-34% of genes listed in AD (N=156) and PD (N=133) Human Disease Ontology databases are expressed at high levels (CPM>50) in two spatially distinct cholinergic cell types. **c, d:** Chord diagrams illustrate abundances (mean CPMs) of the top 10 most expressed AD genes (**c**) and PD genes (**d**) in each of the MS and dorsal CPU cholinergic cell types.

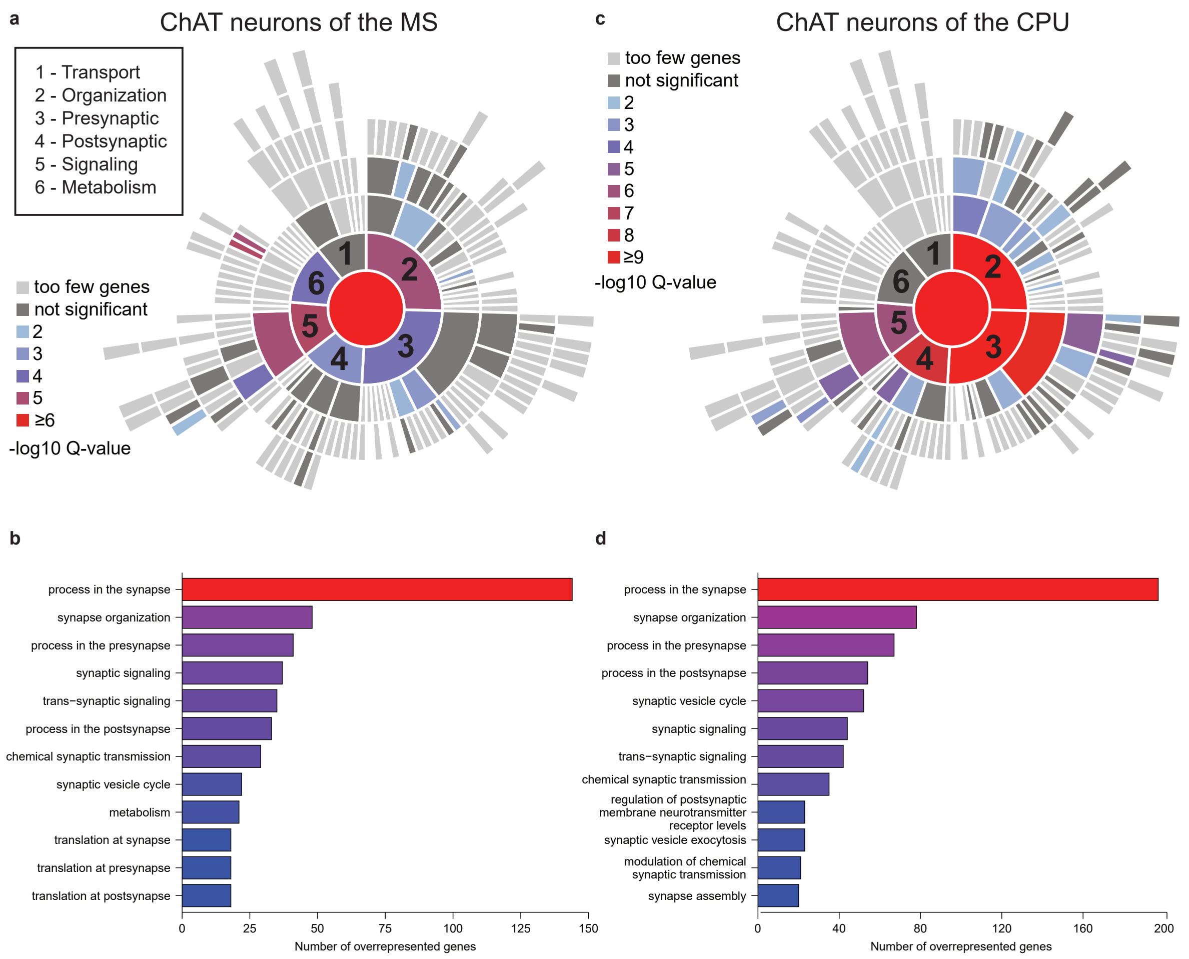

Figure S4: SynGO pathway analysis of differentially expressed genes. Many of the 2,891 transcripts expressed differentially between the MS330 and CPU300 transcriptomes are involved in synaptic functions. Diagrams graphically illustrate the number of genes in different Synaptic Gene Ontology (SynGO) categories. **a, b:** Synaptic genes expressed predominantly in MS cholinergic neurons. **c, d:** Synaptic genes with predominant expression in cholinergic neurons of the dorsal CPU. Specific genes are listed in **Table S3.**

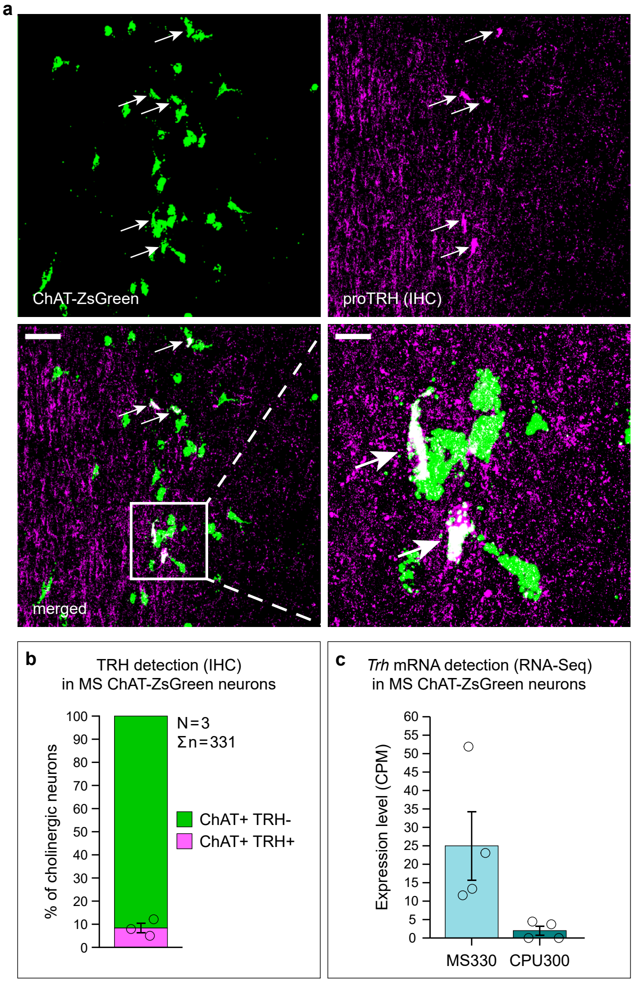

Figure S5: Results of anatomical and gene expression studies indicating TRH cotransmission in a subpopulation of MS cholinergic neurons. a: Detection of proTRH immunoreactivity (magenta) in a subset of MS cholinergic neurons (green; ChAT-ZsGreen signal) with confocal microscopy. Arrows indicate double-labeled cell bodies. **b:** Results of quantitative analysis reveals the immunohistochemical signal for TRH in 8.4±2.1% (mean±SEM) of MS cholinergic neurons. **c:** Selective expression of *Trh* mRNA in the MS330 transcriptome confirms that cholinergic neurons of the MS synthesize *bona fide* proTRH in a cell type specific manner. Scale bars: 50 µm (low power images), 12.5 µm (high power inset).
